# Use of hydrochlorothiazide and risk of skin cancer: a nationwide Taiwanese case–control study

**DOI:** 10.1038/s41416-019-0613-4

**Published:** 2019-11-01

**Authors:** Anton Pottegård, Sidsel Arnspang Pedersen, Sigrun Alba Johannesdottir Schmidt, Chaw-Ning Lee, Chao-Kai Hsu, Tzu-Chi Liao, Shih-Chieh Shao, Edward Chia-Cheng Lai

**Affiliations:** 1 Clinical Pharmacology and Pharmacy, Department of Public health, University of Southern Denmark, Odense, Denmark; 2 Clinical Biochemistry and Pharmacology, Odense University Hospital, Odense, Denmark; 3 Department of Clinical Epidemiology, Aarhus University Hospital, Aarhus, Denmark; 4 Department of Dermatology, Aarhus University Hospital, Aarhus, Denmark; 5 Department of Dermatology, National Cheng Kung University Hospital, College of Medicine, National Cheng Kung University, Tainan, Taiwan; 6 School of Pharmacy, Institute of Clinical Pharmacy and Pharmaceutical Sciences, College of Medicine, National Cheng Kung University, Tainan, Taiwan; 7 Department of Pharmacy, Chang Gung Memorial Hospital, Keelung, Taiwan; 8 Department of Pharmacy, National Cheng Kung University Hospital, Tainan, Taiwan

**Keywords:** Risk factors, Epidemiology, Hypertension

## Abstract

**Background:**

The antihypertensive agent hydrochlorothiazide has been associated with increased risks of non-melanoma skin cancer (NMSC) and possibly some melanoma subtypes. Previous studies were, however, conducted in predominantly Caucasian populations. We therefore examined the association between hydrochlorothiazide and skin cancer risk in an Asian population.

**Methods:**

By using Taiwan’s National Health Insurance Research Database (NHIRD), we conducted three separate case–control studies of lip cancer, non-lip non-melanoma skin cancer and melanoma. Cases (*n* = 29,082) with a first-ever skin cancer diagnoses (2008–2015) were matched 1:10 to population controls. We estimated odds ratios (ORs) associating hydrochlorothiazide use with skin cancer risk by using conditional logistic regression.

**Results:**

Hydrochlorothiazide use showed no overall association with any of the three outcomes: ORs for high cumulative use of HCTZ (≥50,000 mg) were 0.86 (95% CI 0.09–7.81) for lip cancer, 1.16 (95% CI 0.98–1.37) for non-lip NMSC and 1.07 (95% CI 0.65–1.76) for melanoma. There was some evidence of a dose–response pattern for non-lip NMSC, with an OR of 1.66 (95% CI 0.82–3.33) for 100,000–149,999 mg of HCTZ. The null findings were robust across subgroup and sensitivity analyses.

**Conclusion:**

Use of HCTZ appears safe in terms of skin cancer risk in an Asian population.

## Background

The antihypertensive agent hydrochlorothiazide (HCTZ) was recently found to be strongly associated with increased risks of squamous cell carcinoma (SCC) of the lip,^1,2^ non-lip SCC of the skin^3^ and more rare forms of non-melanoma skin cancer (NMSC).^4^ Further, HCTZ is associated with a slightly increased risk of basal cell carcinoma (BCC)^3^ and potentially also certain melanoma subtypes.^5^ These findings support the classification of HCTZ as ‘possibly carcinogenic to humans’ (Group 2B) by the International Agency for Research on Cancer (IARC) in 2013.^6,7^ The underlying mechanism is thought to be the photosensitising properties of HCTZ, increasing users’ susceptibility to ultraviolet radiation (UVR)-induced DNA damage.^8^ In 2018, the Pharmacovigilance Risk Assessment Committee (PRAC) of the European Medicines Agency (EMA) concluded that the reported associations between HCTZ use and SCC and BCC most likely represent causality,^9^ and consequently Summary of Product Characteristics (SmPCs) and package inserts have been updated to reflect this risk, and ‘dear health care professional’ (DHCP) letters have been distributed to inform prescribers across Europe.

Nevertheless, several important questions remain unanswered. Most importantly, these findings need to be replicated in other settings. As previous studies have been carried out in predominantly Caucasian populations, it is unknown whether the risks associated with HCTZ use are generalisable to Asian populations, which have different skin phenotype^10^ and thus a markedly lower baseline risk of skin cancer.^10,11^ We therefore used data from the nationwide Taiwanese health registries to examine the association between HCTZ use and the risk of skin cancer.

## Methods

We performed three separate case–control studies by using the nationwide Taiwanese claims database. By comparing the use of HCTZ among persons diagnosed with skin cancer (cases) with the use among cancer-free population controls, we estimated odds ratios (ORs) associating HCTZ use to the risk of (i) NMSC of the lip, (ii) non-lip NMSC and (iii) melanoma.

### Data sources

Taiwan launched a single-payer mandatory National Health Insurance (NHI) Program on 1 March, 1995. The Taiwan’s National Health Insurance Research Database (NHIRD) derived from claims data of NHI beneficiaries was established for research purposes.^12^ Virtually 100% of Taiwan’s population (~25 million individuals) are enrolled under this programme. The information in NHIRD is stored in different datasheets, including a registry for beneficiaries, ambulatory care claims, inpatient claims, prescriptions dispensed at pharmacies, a registry for medical facilities and a registry for board-certified specialists. We expect full capture of HCTZ use in this database, because the NHI offers reimbursement for all HCTZ-containing medications, and because the database includes most prescription drugs in Taiwan and all drugs reimbursed from inpatient, outpatient and emergency services, and contracted pharmacies.

The Supplementary Information provides complete code lists for cancers, exclusion criteria, drug exposures and covariates.

### Selection of case patients

We included as skin cancer patients (cases) all Taiwanese residents with a first-ever diagnosis of NMSC of the lip or skin or cutaneous melanoma, respectively, recorded in the NHIRD between 1 January, 2008 and 31 December, 2015. We sampled NMSC and melanoma cases individually, i.e., each patient could contribute up to two times (representing a NMSC and a melanoma event) but not as both lip and non-lip NMSC cases. We defined the date of skin cancer diagnosis as the index date. We required cases to have no history of cancer before their diagnosis, except that melanoma cases were allowed to have an NMSC diagnosis. Further, we excluded those with <5 years of continuous residency before sampling and those with any record of (i) organ transplantation, (ii) use of azathioprine or (iii) HIV diagnosis before the index date, as immunosuppressive diseases and treatments are known to increase skin cancer risk.^13–16^

### Population controls

We selected controls by using a risk-set sampling strategy. For each case, we selected ten controls among all Taiwanese residents matched by sex and age (birth year and month) applying the same selection criteria as for cases. Controls were allotted the index dates of their corresponding cases. Persons were eligible as controls before they became cases and could be sampled as controls multiple times. Thereby, the calculated odds ratios (ORs) provide estimates of the incidence rate ratios (IRRs) that would have emerged from a cohort study based on the source population.^17^

### Exposure definition

Based on prescriptions dispensed since 2003, we defined ever-use of HCTZ as at least one filled prescription of a HCTZ-containing drug before the index date, while we defined never-use as no HCTZ-containing prescriptions. We defined high-use of HCTZ as filled prescriptions equivalent to ≥50,000 mg of HCTZ, corresponding to ~6 years of cumulative use (2000 defined daily doses^18^). We disregarded prescriptions filled within 2 years before the index date to allow a reasonable induction period for an effect on NMSC risk and to guard against the possibility that increased medical attention before the cancer diagnosis influenced HCTZ prescribing (‘reverse causation’^19^).

### Covariates

We defined potential confounders based on prescription and hospitalisation data: (a) use of selected drugs with suggested photosensitising properties, including oral retinoids, topical retinoids, tetracycline, macrolides, aminoquinolines and amiodarone;^20–23^ (b) use of drugs with suggested antineoplastic effects, including aspirin, NSAIDs and statins;^7^ (c) hospital diagnoses or disease-specific drugs defining a history of diabetes and chronic obstructive pulmonary disease (COPD); d) total Charlson Comorbidity Index (CCI) scores^24,25^ (0: low; 1–2: medium; or ≥3: high) derived from prevalence of 19 chronic conditions. We defined exposure to each potential confounder drug as two or more prescriptions on separate dates, and hospital histories of each of the selected conditions were defined as a primary or secondary discharge or outpatient diagnosis. For all covariates, we disregarded information within 2 years before the index date.

### Main analyses

We used conditional logistic regression analysis to compute odds ratios (ORs), with 95% confidence intervals (CIs), for each skin cancer outcome associated with HCTZ use adjusted for the predefined potential confounders. In addition, to examine a potential dose–response relationship, we performed analyses stratified according to predefined categories of cumulative HCTZ use: 1–9999, 10,000–24,999, 25,000–49,999, 50,000–74,999, 75,000–99,999, 100,000–149,999, 150,000–199,999 and ≥200,000 mg. We used never-use of HCTZ as the reference group for all analyses. We analysed lip NMSC, non-lip NMSC and melanoma independently.

### Secondary and sensitivity analyses

We performed a number of preplanned supplementary and sensitivity analyses.

First, we performed subgroup analyses according to age and sex, and with restriction to specific subsets of the study population: never-use of other photosensitising drugs (as defined above), low comorbidity (CCI score = 0), no history of diabetes (as patients with diabetes have been shown to have an increased risk of cancer^26^), no history of actinic keratosis (associated with UVR exposure and considered a precursor of NMSC^27^) and no history of atopic dermatitis and psoriasis (associated with UVR exposure and possibly associated with NMSC risk^28,29^).

Secondly, we attempted to address the limitation that the database used did not allow for identification of subtypes of NMSC. The two dominant subtypes of NMSC are BCC and SCC, while other subtypes such as Merkel cell carcinoma and malignant adnexal skin tumours are rare. As HCTZ has been more strongly associated with SCC than BCC,^3^ the results for the overall NMSC risk will be an average of the risk of the two subtypes, weighed by their relative frequency. To contextualise the findings, we therefore sought to identify the SCC:BCC ratio by using the electronic health records from National Cheng-Kung University Hospital, which is the largest medical centre in Southern Taiwan. Two dermatologists retrieved the data from 1 January, 2008 to 31 December, 2015 and confirmed SCC and BCC independently by the pathology diagnosis. In this assessment, patients with doubtful pathological diagnosis, Bowen’s disease and metastatic squamous cell carcinoma from other organs were excluded.

Third, we repeated the main analyses for the thiazide-like diuretic indapamide. Other comparable thiazides (e.g., bendroflumethiazide) and thiazide-like diuretics (e.g., chlorthalidone) were not used in Taiwan during the study period. For comparison, we also performed analyses for antihypertensives with indications similar to those for thiazides (i.e., primarily mild- to-moderate hypertension) but with no suspicion of an increased risk of skin cancer (negative control exposures), including angiotensin-converting enzyme inhibitors, angiotensin II antagonists and group 2 calcium-channel blockers. In the analyses for other diuretics and non-diuretic antihypertensives, associations were adjusted for HCTZ use.

Fourth, we excluded ever-users of amiloride from the main analyses to obtain risk estimates for NMSC with HCTZ use exclusive of amiloride. We did this analysis to obtain estimates outside the amiloride–HCTZ combination, which is the dominant source of HCTZ exposure among HCTZ high users.

Finally, we repeated the main analyses by varying the lag time between 0 and 5 years (in 6-month increments).

We performed all analyses in SAS 9.4 version software (SAS Institute, Cary, NC).

## Results

We identified 187 lip cancer cases, 23,703 non-lip NMSC cases and 5192 melanoma cases (presented overall in Table 1 and stratified in the Supplementary Table). Median age was 69 years (interquartile range [IQR], 53–80 years) and 51% were men. Cases of lip and non-lip NMSC were generally older (median age 66 and 72 years, respectively) compared with melanoma cases (median age 51 years). Cases had a higher comorbidity level than population controls and also a higher use of photosensitising drugs, in particular topical retinoids (1.83 vs. 0.94%).

**Table 1:** Characteristics of skin cancer cases and matched population controls

	Cases	Controls
	(*n* = 29082)	(*n* = 290820)
Age, median (IQR)	69 (53–80)	69 (53–80)
Male gender	14,881 (51.17)	148,810 (51.17)
Skin cancer diagnosis		
Lip cancer	187 (0.64)	NA
Non-lip NMSC	23,703 (81.50)	NA
Melanoma	5192 (17.85)	NA
Use of HCTZ		
Never-use	22,763 (78.27)	235,091 (80.84)
Ever-use	6319 (21.73)	55,729 (19.16)
High-use^a^	189 (0.65)	1619 (0.56)
Use of photosensitising drugs		
Topical retinoids	531 (1.83)	2738 (0.94)
Oral retinoids	24 (0.08)	75 (0.03)
Tetracycline	197 (0.68)	1237 (0.43)
Macrolides	1056 (3.63)	9142 (3.14)
Aminoquinolines	191 (0.66)	1540 (0.53)
Amiodarone	592 (2.04)	4756 (1.64)
Other drug use		
Aspirin	6543 (22.5)	59,260 (20.38)
Non-aspirin NSAID	21,584 (74.22)	191,687 (65.91)
Statins	5037 (17.32)	44,715 (15.38)
Diagnoses		
Diabetes	5265 (18.1)	46,476 (15.98)
COPD	1103 (3.79)	9585 (3.30)
CCI score		
0	8057 (27.70)	112,530 (38.69)
1	8909 (30.63)	69,396 (23.86)
2	5698 (19.59)	44,563 (15.32)
≥3	6418 (22.07)	64,331 (22.12)

*HCTZ* hydrochlorothiazide, *IQR* interquartile range, *NMSC* non-melanoma skin cancer, *CCI* Charlson comorbidity index

^a^High-use = high cumulative dose (≥50,000 mg of HCTZ)

For all three outcomes, ever-use of HCTZ was common among both cases (22%) and controls (19%), while high-use was limited (0.65 and 0.56%, respectively). Overall, HCTZ use showed limited associations to any of the three outcomes (Table 2). For lip cancer, analyses were hindered by low numbers, with high-use of HCTZ returning a fully adjusted OR of 0.86 (95% CI 0.09–7.81) and dose–response analyses precluded due to the limited statistical power. For non-lip NMSC, ever-use and high-use of HCTZ showed slightly increased risks in the minimally adjusted models (OR 1.20 and 1.25, respectively). These estimates were partially attenuated in the fully adjusted models, with high-use yielding an OR of 1.16 (95% CI 0.98–1.37). There was some evidence of a dose–response pattern, with estimates increasing with cumulative use beyond 75,000 mg, reaching an OR of 1.66 (95% CI 0.82–3.33) with use of 100,000–149,999 mg of HCTZ, although the statistical precision of these estimates was limited. For melanoma, high-use of HCTZ yielded an OR of 1.07 (95% CI 0.65–1.76) with limited evidence of a dose–response pattern.

**Table 2:** Association between cumulative exposure to hydrochlorothiazide and risk of lip cancer, non-lip non-melanoma skin cancer and melanoma

Subgroup	Cases	Controls	Adjusted OR^a^	Adjusted OR^b^
*Lip cancer*^**c**^				
Nonuse	143 (76.47)	1524 (81.5)	1.00 (ref.)	1.00 (ref.)
Ever-use	44 (23.53)	346 (18.5)	1.41 (0.96–2.06)	1.18 (0.79–1.76)
High-use (≥50,000 mg)	*N* < 5	*N* < 5	1.58 (0.18–13.62)	0.86 (0.09–7.81)
*Non-lip non-melanoma skin cancer*				
Nonuse	18,121 (76.45)	187,793 (79.23)	1.00 (ref.)	1.00 (ref.)
Ever-use	5582 (23.55)	49237 (20.77)	1.20 (1.16–1.24)	1.10 (1.06–1.14)
High-use (≥50,000 mg)	167 (0.70)	1440 (0.61)	1.25 (1.06–1.48)	1.16 (0.98–1.37)
Cumulative dose:				
1–9999 mg	4009 (16.91)	34993 (14.76)	1.20 (1.16–1.25)	1.11 (1.07–1.15)
10,000–24,999 mg	912 (3.85)	8108 (3.42)	1.18 (1.10–1.27)	1.08 (1.01–1.17)
25,000–49,999 mg	494 (2.08)	4696 (1.98)	1.12 (1.01–1.23)	1.02 (0.93–1.13)
50,000–74,999 mg	128	1132	1.20 (0.99–1.45)	1.11 (0.92–1.35)
75,000–99,999 mg	29	227	1.45 (0.97–2.17)	1.31 (0.87–1.97)
100,000–149,999 mg	10	71	1.70 (0.85–3.41)	1.66 (0.82–3.33)
150,000–199,999 mg	*N* < 5	*N* < 5	–	–
≥200,000 mg	*N* < 5	*N* < 5	–	–
*Melanoma*				
Nonuse	4499 (86.65)	45774 (88.16)	1.00 (ref.)	1.00 (ref.)
Ever-use	693 (13.35)	6146 (11.84)	1.18 (1.08–1.3)	0.90 (0.82–0.99)
High-use (≥50,000 mg)	21 (0.40)	172 (0.33)	1.29 (0.81–2.07)	1.07 (0.65–1.76)
Cumulative dose:				
1–9999 mg	508 (9.78)	4419 (8.51)	1.20 (1.08–1.33)	0.93 (0.83–1.03)
10,000–24,999 mg	108 (2.08)	1025 (1.97)	1.10 (0.89–1.35)	0.89 (0.71–1.11)
25,000–49,999 mg	56	530	1.10 (0.83–1.47)	0.89 (0.65–1.2)
50,000–74,999 mg	20	138	1.51 (0.93–2.47)	1.22 (0.73–2.06)
75,000–99,999 mg	*N* < 5	*N* < 5	–	–
100,000–149,999 mg	*N* < 5	*N* < 5	–	–
150,000–199,999 mg	*N* < 5	*N* < 5	–	–
≥200,000 mg	*N* < 5	*N* < 5	–	–

*OR* odds ratio

A dash (–) denotes ORs that could not be estimated due to small numbers

^a^Adjusted for age, gender and calendar time (by risk-set matching and the conditional analysis)

^b^Fully adjusted model, i.e., additionally adjusted for (a) use of topical retinoids, oral retinoids, tetracycline, macrolides, aminoquinolines or amiodarone; (b) aspirin, non-aspirin non-steroidal anti-inflammatory drugs (NSAIDs) or statins; (c) history of diabetes, or chronic obstructive pulmonary disease (COPD); (d) Charlson Comorbidity Index (CCI) score (0: low; 2: medium; ≥3: high)

^c^The low number of cases precluded detailed dose–response analyses of lip cancer

In subgroup analyses and upon exclusion of different risk groups, the overall estimates showed little deviation, although with limited statistical power in most of the subgroups (Table 3).

**Table 3:** Associations between high cumulative use of hydrochlorothiazide (≥50,000 mg) and risk of lip cancer, non-lip non-melanoma skin cancer and melanoma, specified by patient subgroups

	Lip cancer (OR; 95% CI)^a^	Non-lip NMSC (OR; 95% CI)^a^	Melanoma (OR; 95% CI)^a^
All	0.86 (0.09–7.81)	1.16 (0.98–1.37)	1.07 (0.65–1.76)
*Age groups*			
<50 years	–	–	–
50–60 years	–	0.66 (0.20–2.16)	1.03 (0.11–10.07)
60–75 years	–	1.45 (1.07–1.97)	0.93 (0.37–2.32)
75+ years	1.13 (0.11–11.2)	1.10 (0.89–1.35)	1.12 (0.61–2.08)
*Sex*			
Male	2.72 (0.19–38.3)	1.29 (1.02–1.64)	1.08 (0.53–2.21)
Female	–	1.05 (0.83–1.33)	1.06 (0.53–2.14)
*Other subgroups*			
No history of use of photosensitising drugs	1.37 (0.13–14.08)	1.16 (0.97–1.39)	1.16 (0.69–1.96)
CCI score = 0	–	1.07 (0.54–2.11)	–
No history of diabetes	–	1.09 (0.86–1.39)	1.06 (0.55–2.05)
No history of psoriasis or atopic dermatitis	0.86 (0.09–7.81)	1.16 (0.98–1.37)	1.07 (0.65–1.76)
No history of actinic keratosis	0.84 (0.09–7.71)	1.14 (0.96–1.36)	1.03 (0.62–1.71)

*NMSC* non-melanoma skin cancer, *OR* odds ratio, *CCI* Charlson comorbidity index

A dash (–) denotes ORs that could not be estimated due to small numbers

^a^Adjusted for (a) age, gender and calendar time (by risk-set matching and the conditional analysis); (b) use of topical retinoids, oral retinoids, tetracycline, macrolides, aminoquinolines or amiodarone; (c) aspirin, non-aspirin non-steroidal anti-inflammatory drugs (NSAIDs) or statins; (d) history of diabetes or chronic obstructive pulmonary disease (COPD); (e) Charlson Comorbidity Index (CCI) score (0: low; 2: medium; or ≥3: high)

The assessment of the SCC:BCC ratio identified 812 eligible cases of NMSC, of which 290 (36%) were classified as SCC and 522 (64%) were classified as BCC.

Analyses of the thiazide-like diuretic indapamide (ever-use) showed no association to lip cancer (OR 0.92), NMSC (0.98) or melanoma (OR 0.81). The planned analyses of other antihypertensives as negative controls were abandoned due to the lack of associations observed in the main analyses.

Finally, the sensitivity analyses excluding amiloride ever-users or applying lag times varying from 0 to 5 years supported the main results (data not shown).

## Discussion

Leveraging nationwide Taiwanese health data, we provide the first assessment of skin cancer risk with use of HCTZ in a predominantly Asian population. Overall, in this population, use of HCTZ appears safe, with no evidence of a clinically relevant increased risk of any of the skin cancer types investigated. While there was some evidence of an increased relative risk of non-lip NMSC with high cumulative use of HCTZ, these estimates were based on a low number of exposed cases.

The primary strength of our analysis is the use of the nationwide data source of NHIRD,^12^ allowing almost complete capture of 25 million individuals’ prescriptions and skin cancer history. Some limitations do also need to be considered. First, prescription data are in NHIRD only available since 2003. As we sampled cases and controls from 2008 to 2015, we had 5–13 years of prescription history for subjects in the study. This censoring of exposure data will misclassify some individuals in terms of cumulative HCTZ dose filled. While inclusion of HCTZ ever-users in the never-use reference category is most likely a negligible problem, there might be more substantial misclassification of longer-term HCZT users. While this could obscure analyses of dose–response patterns, by underestimating use among those with substantial use before 2003, it is less of a concern in the face of overall null findings. Second, the follow-up period of down to 5 years might be too short to detect an increased risk of skin cancer with HCTZ use, which could attenuate or mask a true increased risk. However, as many HCTZ users will likely have used HCTZ before 2003, we do not expect this to be the sole explanation of the negative finding. Third, the quality of skin cancer diagnoses recorded in NHIRD has not been validated. However, validation studies have generally found high positive predictive values for major diseases^12^ as well as for cancer overall.^30^ Fourth, the lack of the ability to distinguish between NMSC as well as melanoma subtypes hinders the interpretation of the study’s findings. The increased risk of skin cancer with use of HCTZ observed in Caucasians is markedly more pronounced for SCC compared with BCC skin cancer,^3^ and thus the combined endpoint of ‘any NMSC‘ might mask a true increased risk of SCC NMSC. The analysis of the SCC/BCC ratio showed that SCCs constituted one-third of Taiwanese NMSC cases. This, in combination with the general finding of null associations, makes it unlikely that any major increased risk of SCC has been overlooked. This is further supported by the lack of an association for lip cancer, known to comprise mainly SCCs.^31^ Fifth, the generally limited statistical power made interpretations difficult, in particular for subgroup analyses. Importantly, however, the limited power, despite use of a data source covering 25 million subjects, is due to the very low overall risk of skin cancer in Asian populations. As such, even under the assumption that the slightly increased risks seen with long-term HCTZ use in this study represent a causal association, or even if one were to cautiously use the estimate corresponding to the upper limit of the 95% CIs, as could be considered more relevant for safety outcomes, the increased risk of skin cancer with use of HCTZ is, in an Asian population, most likely negligible in absolute terms.

The recent studies on HCTZ use and skin cancer risk, all carried out in Caucasian populations, found very strong associations, in particular for SCC of the lips and skin, where estimates increased in a dose-dependent manner up to ORs of 7–8 with use of > 200,000 mg of HCTZ.^1–3^ This contrasts the null findings of this study. Differences in adverse effects of medications across different ethnicities are not an unknown phenomenon. As a prominent example, the occurrence of Stevens–Johnson syndrome and toxic epidermal necrolysis with use of carbamazepine was found to be very closely linked to the HLA-B*15:02 allele among Taiwanese residents,^32^ which was later replicated in Chinese, Indian, Malaysian and Chinese–American residents.^33^ However, this allele is largely absent in non-Asian individuals.^33^ The lack of an association between HCTZ use and skin cancer in an Asian population can have multiple explanations. First, persons of Asian descent typically have Fitzpatrick skin type III or IV, where melanin content is higher, and DNA repair mechanisms are possibly more efficient^10^ than in more fair skinned Northern European populations. It is thus possible that skin phenotype acts as an effect modifier, providing Asian populations with natural protection against the photosensitising effects of HCTZ. Second, the patterns of HCTZ use in the Taiwanese population differ from those previously reported in the Danish population,^3^ with generally lower doses and consequently very few long-term users. It might be speculated that a threshold effect exists, such that a low daily intake of HCTZ might not be associated with skin cancer risk, or at least not to the same extent as that of higher daily HCTZ intake. Third, cultural differences in sun exposure and behaviour might also play a role. Fair skin is traditionally considered beautiful in Asia, in particular among women. This, as well as the tropical/subtropical climate of Taiwan, makes many people wear hats or use umbrellas while outdoors. Such sun-protective behaviours might mitigate the potential harm from photosensitising compounds, including HCTZ. While it is likely that all of the three above-mentioned explanations contribute to the null finding of this analysis, we find it most likely that the differences in skin type is the primary driver of the lack of effect of HCTZ on skin cancer risk in the Taiwanese population.

In conclusion, we found limited evidence of an increased risk of skin cancer with use of HCTZ in a Taiwanese population. While some limitations might have hindered detection of minor excess risks, including lack of differentiation of NMSC subtypes, left censoring of exposure data and relatively limited follow-up, the study findings do not support that skin cancer should be considered a side effect to HCTZ use in an Asian population, in particular when considering the low absolute risks of skin cancer in this population.

## Supplementary information

SUPPLEMENTAL INFORMATION

## Data Availability

The data that support the findings of this study are available from Taiwan’s National Health Insurance Research Database (NHIRD). Restrictions apply to the availability of these data, which were used under license for this study.
