# Supplementary material for: Use of hydrochlorothiazide and risk of skin cancer: a nationwide Taiwanese case–control study
Source: Br J Cancer. 2019 Nov 1;121(11):973–8. doi: 10.1038/s41416-019-0613-4 (PMC6889460; doi:10.1038/s41416-019-0613-4)
Supplement: Supplementary file 1 — SUPPLEMENTAL INFORMATION [file 41416_2019_613_MOESM1_ESM.docx]

**Supplementary Information – Codes and definitions**

| **Cancer definitions** | |  |
| --- | --- | --- |
| Non-melanoma skin cancer of the lip | *ICD-9* | 1730 |
| Non-lip non-melanoma skin cancer | *ICD-9* | 1731-1739 |
| Cutaneous melanoma | *ICD-9* | 1720-1729 |
|  |  |  |
| **Exclusion criteria** | |  |
| Any cancer, except skin cancer | *ICD-9* | 14000-20891 (except 1720-1739) |
| Organ transplant | *ICD-9* | V42 |
|  | *NCSP-code* | KFQA, KFQB, KGDG, KJJC, KJLE, KKAS |
| Azathioprine | *ATC code* | L04AX01 |
| HIV | *ICD-9* | 042 |
|  |  |  |
|  | |  |
| **Use of drugs (≥ 2 fillings prior to index date)** | |  |
| HCTZ | *ATC code* | C03AA03 |
| HCTZ and potassium | *ATC code* | C03AB |
| HCTZ and amilorid | *ATC code* | C03EA01 |
| HCTZ and AT2 antagonist | *ATC code* | C09DA01, C09DA03, C09DA04, C09DA06, C09DA07, C09DA08 |
| HCTZ and ACE inhibitor | *ATC code* | C09BA02, C09BA03, C09BA05, |
| HCTZ/calcium antagonist/AT 2 antagonist | *ATC code* | C09DX01 |
| HCTZ/metoprolol | *ATC code* | C07BB02 |
| Topical retinoids | *ATC code* | D10AD |
| Oral retinoids | *ATC code* | D05BB D10BA01 |
| Tetracycline | *ATC code* | J01AA07 |
| Macrolides | *ATC code* | J01FA |
| Aminoquinolines | *ATC code* | P01BA |
| Amiodarone | *ATC code* | C01BD01 |
| Low-dose aspirin | *ATC code* | B01AC06, B01AC30, N02BA01, and N02BA51 |
| Non-aspirin NSAIDs | *ATC code* | M01A excl. M01AX |
| Statins | *ATC code* | C10AA |
| **Prior diagnoses (diagnostic code or drug marker)** | | |
| Diabetes | ICD-9 | 250, 648 |
|  | *ATC code* | A10 |
| COPD | ICD-9 | 490-496 |
|  | *ATC code* | R03BB |
| Actinic keratosis | *ICD-9* | 7020 |
| Psoriasis | *ICD-9* | 6960, 6961 |
|  | *ATC code* | D05AX |
| Atopic Dermatitis | *ICD-9* | 6918 |
| NOTES:  ICD = International Classification of Disease  ATC = Anatomical Therapeutic Chemical  NCSP = Nordic Classification of Surgical procedures | | |

COPD= chronic obstructive pulmonary disease

**Supplementary Table**Characteristics of skin cancer cases and matched population controls

|  | **Lip NMSC** | | **Non-lip NMSC** | | **Melanoma** | |
| --- | --- | --- | --- | --- | --- | --- |
|  | Cases | Controls | Cases | Controls | Cases | Controls |
|  | (n=187) | (n=1,870) | (n=23,703) | (n=237,030) | (n=5,192) | (n=51,920) |
| Age, median (IQR) | 66 (52-78) | 66 (52-78) | 51 (24-72) | 72 (57-81) | 51 (24-72) | 51 (24-72) |
| Male gender | 96 (51.34) | 960 (51.34) | 12257 (51.71) | 122560 (51.71) | 2528 (48.69) | 25290 (48.71) |
| Use of HCTZ |  |  |  |  |  |  |
| Never-use | 143 (76.47) | 1524 (81.5) | 18121 (76.45) | 187793 (79.23) | 4499 (86.65) | 45774 (88.16) |
| Ever-use | 44 (23.53) | 346 (18.5) | 5582 (23.55) | 49237 (20.77) | 693 (13.35) | 6146 (11.84) |
| High-use^*^ | NA | NA | 167 (0.7) | 1440 (0.61) | 21 (0.4) | 172 (0.33) |
| Use of photosens. drugs |  |  |  |  |  |  |
| Topical retinoids | NA | 20 (1.07) | 407 (1.72) | 2176 (0.92) | 118 (2.27) | 542 (1.04) |
| Oral retinoids | NA | 0 (0) | 16 (0.07) | 66 (0.03) | 8 (0.15) | 9 (0.02) |
| Tetracycline | NA | 7 (0.37) | 156 (0.66) | 1023 (0.43) | 40 (0.77) | 207 (0.4) |
| Macrolides | NA | 54 (2.89) | 852 (3.59) | 7302 (3.08) | 200 (3.85) | 1786 (3.44) |
| Aminoquinolines | NA | 11 (0.59) | 172 (0.73) | 1330 (0.56) | 18 (0.35) | 199 (0.38) |
| Amiodarone | NA | 23 (1.23) | 522 (2.2) | 4228 (1.78) | 66 (1.27) | 505 (0.97) |
| Other drug use |  |  |  |  |  |  |
| Aspirin | 45 (24.06) | 378 (20.21) | 5745 (24.24) | 52285 (22.06) | 753 (14.5) | 6597 (12.71) |
| Non-aspirin NSAID | 147 (78.61) | 1229 (65.72) | 17468 (73.7) | 155642 (65.66) | 3969 (76.44) | 34816 (67.06) |
| Statins | 42 (22.46) | 311 (16.63) | 4385 (18.5) | 39393 (16.62) | 610 (11.75) | 5011 (9.65) |
| Diagnoses |  |  |  |  |  |  |
| Diabetes | 43 (22.99) | 294 (15.72) | 4629 (19.53) | 40860 (17.24) | 593 (11.42) | 5322 (10.25) |
| COPD | 8 (4.28) | 49 (2.62) | 962 (4.06) | 8373 (3.53) | 133 (2.56) | 1163 (2.24) |
| CCI-score |  |  |  |  |  |  |
| 0 | 65 (34.76) | 735 (39.3) | 7974 (33.64) | 84700 (35.73) | 18 (0.35) | 27095 (52.19) |
| 1 | 52 (27.81) | 471 (25.19) | 6264 (26.43) | 57433 (24.23) | 2593 (49.94) | 11492 (22.13) |
| 2 | 34 (18.18) | 294 (15.72) | 4292 (18.11) | 38900 (16.41) | 1372 (26.43) | 5369 (10.34) |
| ≥3 | 36 (19.25) | 370 (19.79) | 5173 (21.82) | 55997 (23.62) | 1209 (23.29) | 7964 (15.34) |

NOTES:

NA = Not available (due to count < 5)

HCTZ = Hydrochlorothiazide

IQR = Interquartile range

NMSC = Non-melanoma skin cancer

CCI = Charlson comorbidity index

*High-use = High cumulative dose (≥50,000 mg of HCTZ)
